# How much oxygen in adult cardiac arrest?

**DOI:** 10.1186/s13054-014-0555-4

**Published:** 2014-10-07

**Authors:** Antonio Maria Dell’Anna, Irene Lamanna, Jean-Louis Vincent, Fabicpro Silvio Taccone

**Affiliations:** Department of Intensive Care, Erasme Hospital, Université Libre de Bruxelles, Belgium, Route de Lennik 808, 1070 Brussels, Belgium

## Abstract

Although experimental studies have suggested that a high arterial oxygen pressure (PaO_2_) might aggravate post-anoxic brain injury, clinical studies in patients resuscitated from cardiac arrest (CA) have given conflicting results. Some studies found that a PaO_2_ of more than 300 mm Hg (hyperoxemia) was an independent predictor of poor outcome, but others reported no association between blood oxygenation and neurological recovery in this setting. In this article, we review the potential mechanisms of oxygen toxicity after CA, animal data available in this field, and key human studies dealing with the impact of oxygen management in CA patients, highlighting some potential confounders and limitations and indicating future areas of research in this field. From the currently available literature, high oxygen concentrations during cardiopulmonary resuscitation seem preferable, whereas hyperoxemia should be avoided in the post-CA care. A specific threshold for oxygen toxicity has not yet been identified. The mechanisms of oxygen toxicity after CA, such as seizure development, reactive oxygen species production, and the development of organ dysfunction, need to be further evaluated in prospective studies.

## Introduction

Sudden cardiac arrest (CA) is the leading cause of death among adults worldwide [1,2]. In most patients, attempts at cardiopulmonary resuscitation (CPR) remain ineffective and spontaneous cardiac activity cannot be restored [3]. Among those patients who do achieve return of spontaneous circulation (ROSC), there are two key periods when death may occur: early (during the first three days), usually because of recurrent CA or severe cardiovascular failure resulting in multiple organ failure (MOF), and late (beyond day 3), usually secondary to withdrawal of life-sustaining therapies in the absence of neurological recovery [4]. Although several interventions, including target temperature management (TTM), have been introduced into the post-CA care of these patients [5,6], conflicting results have been obtained [7], and these approaches are not sufficient to prevent the deleterious consequences of brain ischemia in all patients. During the post-CA care, secondary brain insult must be avoided [8] and optimization of brain oxygenation is likely to be an important component of brain recovery. The restoration of adequate systemic hemodynamics is a prerequisite to provide adequate cerebral blood flow in CA patients [9,10], but brain oxygenation is also determined by the arterial oxygen content. Arterial oxygen pressure (PaO_2_) itself may influence brain cellular oxygen supply; if hypoxemia (that is, PaO_2_ of less than 60 mm Hg) is associated with poor outcomes after CA [11], a high PaO_2_ may also be detrimental in a vulnerable brain, as suggested in patients with traumatic brain injury or stroke [12,13]. The aims of this article are to review the potential mechanisms of oxygen toxicity after CA and to discuss the clinical impact of oxygen management on post-CA care.

## Post-cardiac arrest syndrome: the role of oxygen

**Figure 1:**
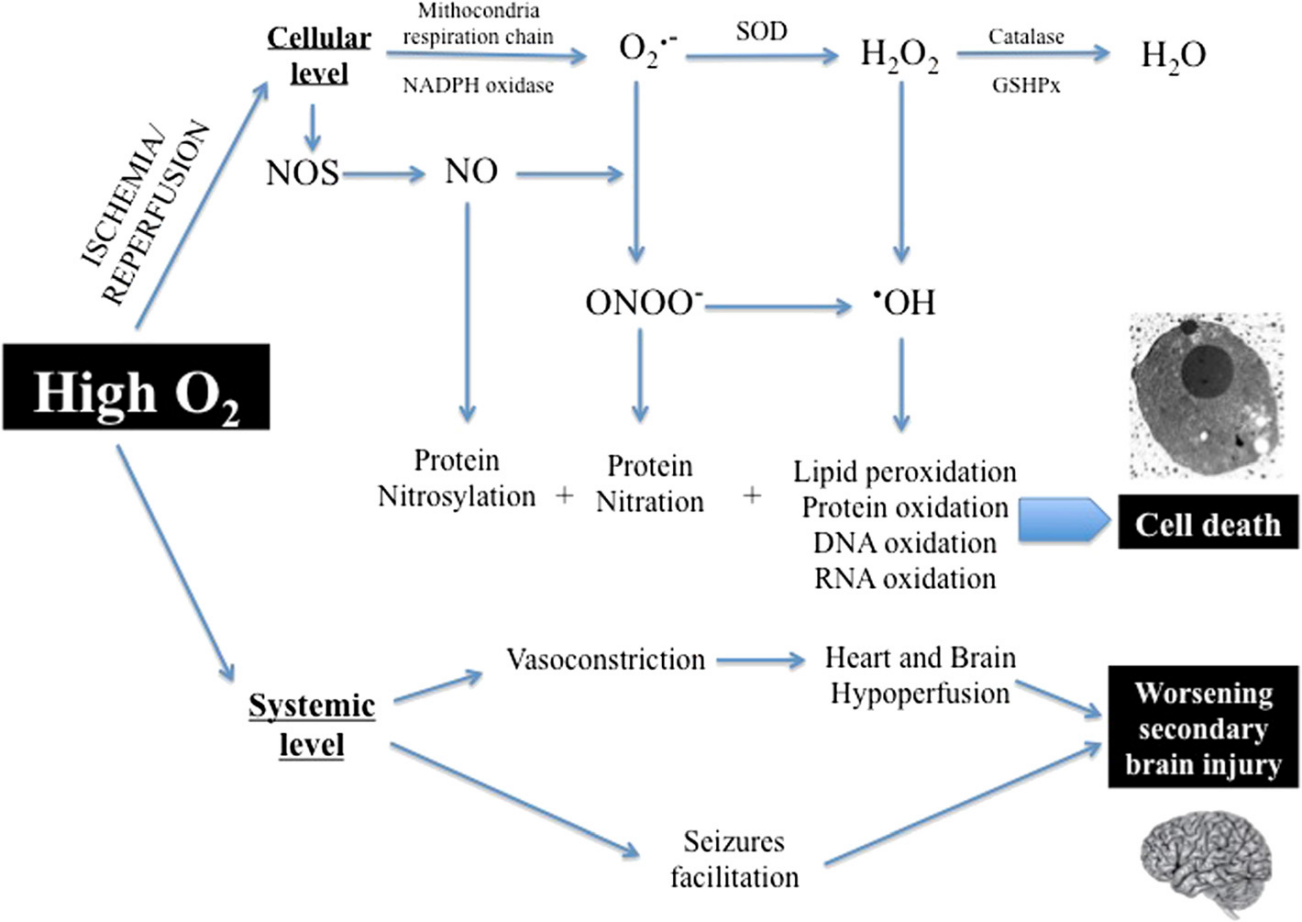
**Summary of cellular and systemic effects of high oxygen (O**_**2**_ **) concentrations.** H_2_O, water; H_2_O_2_, hydrogen peroxide; NO, nitric oxide; NOS, nitric oxide synthase; O_2_^• −^, superoxide ion; ^•^OH, hydroxide ion; ONOO^−^, peroxynitrite ion.

Post-cardiac arrest syndrome (PCAS) is a complex phenomenon, which shares several features with septic shock [7,14]. In particular, PCAS includes a systemic inflammatory response that can be triggered by the ischemia-reperfusion injury and also specific precipitating events, such as concomitant infections or heart disease. Moreover, PCAS can contribute to brain injury and myocardial dysfunction and can rapidly lead to MOF. The primary ischemia-reperfusion injury [15] activates various intracellular pathways, promoting ion concentration disequilibrium with increased intracellular levels of inorganic phosphate, lactate, and H^+^, and resulting in an influx of calcium into the cell [16], which aggravates mitochondrial dysfunction and eventually leads to programmed cellular death (apoptosis). After reperfusion has occurred, other mediators, including superoxide (O_2_^−^), peroxynitrite (NO_2_^−^), hydrogen peroxide (H_2_O_2_), and hydroxyl radicals (OH^−^), contribute to worsen cellular function by oxidizing and damaging numerous cellular components [17] (Figure 1). These reactive oxygen species (ROS) then have a central role in initiating and enhancing the post-ischemic damage [15]. Indeed, supra-normal oxygen concentrations in this context may further stimulate ROS production and contribute to worsen cellular function in a setting of impaired mitochondrial function and impaired oxygen utilization. Moreover, some other systemic detrimental effects of hyperoxemia have been known for many years [18-20]. Hyperoxemia causes systemic and coronary vasoconstriction, which can decrease cardiac output and induce myocardial ischemia. In some experimental models of global cerebral ischemia, hyperoxemia has been shown to be detrimental to the brain, probably also because of its vasoconstrictor effects [21,22]. Hyperoxemia may also provoke or exacerbate seizures, which could aggravate brain injury [23,24].

## Oxygen therapy after cardiac arrest: animal studies

Several studies have assessed the effects of administering high oxygen concentrations in experimental models of CA. Pilcher and colleagues [25] recently reviewed studies that evaluated the role of different oxygenation strategies - that is, one with 100% inspired oxygen fraction (FiO_2_) and the other with lower FiO_2_ as a control - after ROSC. Six studies including 95 animals of different species were included in their final meta-analysis; in general, administration of high FiO_2_ (100%) for 1 hour after ROSC resulted in a worse neurological outcome, as assessed by a neurological deficit score, than other FiO_2_ values. Four of the five studies that assessed histological damage reported a significantly higher neuronal injury with high FiO_2_; cerebral metabolic function was also more altered in the high FiO_2_ group. The extrapolation of such findings to humans, however, may be misleading. First, in all of these experimental studies, the animals were already mechanically ventilated before CA, and this is not usually the case in humans; also, control animals did not all receive the same FiO_2_ (ranging from 21% to higher levels based on PaO_2_ values), and the intervention period was rather short (that is, 1 hour). Moreover, different models of CA were employed (that is, asphyxia versus electrical-induced ventricular fibrillation), with shorter durations of CPR than in humans, and in some articles clear PaO_2_ targets were aimed at, whereas in others only FiO_2_ was modified without looking at PaO_2_ values. In addition, no potential neuroprotective therapy, such as TTM, was provided, so that its influence cannot be ascertained. In summary, animal data have highlighted a clear correlation between the application of high FiO_2_ after CA and poor neurological recovery. Nevertheless, experimental models are quite remote from the human setting, so that the observations cannot be readily translated to humans in the post-CA management.

## Oxygen after cardiac arrest: human studies

Two studies reported conflicting results regarding oxygen management during the early phase of CA (Table 1). In 145 out-of-hospital CA patients, Spindelboeck and colleagues [26] observed that high PaO_2_ (more than 300 mm Hg) levels during CPR were associated with higher rates of ROSC and intact neurological survival when compared with low (PaO_2_ of less than 60 mm Hg) and normal PaO_2_. In contrast, Kuisma and colleagues [27] randomly assigned 28 patients to receive either 30% or 100% FiO_2_ for 1 hour after ROSC; five patients (36%) in the group with lower FiO_2_ required an increase in FiO_2_ to 40% because oxygen saturation fell below 95%, according to a standardized protocol. The primary endpoints were biomarker levels of brain injury - that is, neuron-specific enolase (NSE) and S100B - at 24 and 48 hours after ROSC, with no differences in absolute values between the two groups overall. However, in the subgroup of patients who did not undergo TTM, those receiving lower FiO_2_ had lower NSE levels at 24 hours than the other patients (7.6 ± 4.2 versus 13.5 ± 9.6 μg/mL, *P* = 0.049). Thus, one may argue that high PaO_2_ during CPR reflects better lung function or better quality CPR or both, with higher blood flow and better tissue oxygenation (indeed all patients received FiO_2_ of 100%), whereas administration of high FiO_2_ immediately after ROSC could enhance brain injury. Importantly, the retrospective nature of the study by Spindelboeck and colleagues [26] may have limited the analysis of all factors possibly affecting outcome (that is, quality of CPR, comorbidities, and so on). Also, blood gas analysis samples were drawn within 60 minutes from CPR initiation, and it is difficult to compare patients analyzed in the early CPR with those included in the later phase, who may have developed pulmonary injury with reduced PaO_2_ due to prolonged resuscitation itself. It is difficult to determine the role of oxygen levels during CPR *per se* on neurological outcome of CA patients because they may be a surrogate marker of resuscitation performance or better cardiorespiratory status or both. Moreover, brain injury is a continuous process, and the time course of PaO_2_ may more significantly influence neurological outcome in these patients.

**Table 1:** **Summary of clinical studies evaluating the role of oxygen concentrations on outcome after cardiac arrest**

**Reference**	**Type**	**Patients (period)% of HO**	**OHCA**	**Definition HO**	**Evaluation**	**TH (% treated)**	**Cutoff** ^**a**^	**Outcome**	**Main results**
During CPR									
Spindelboeck *et al*. [26]	R	145 (8 years) 14%	100%	>300 mm Hg	During CPR	NA	NR	In-hospital CPC	Higher rate of hospital admission in hyperoxemic patients
After ROSC									
Kuisma *et al*. [27]	RCT	28 (NA) 50%	100%	1 hour of ventilation at FiO_2_ 100%	24- and 48-hour	No (50%)	No	NSE and S100B	No difference in biomarkers of brain injury
Post-CA care (ICU stay)									
Kilgannon *et al*. [28]	R/D	6,326 (5 years) 18%	43%	First ABG >300 mm Hg	24-hour	NR ≈ 6%	No	In-hospital death	Increased hospital mortality in hyperoxemic patients
								Neurological function	
Kilgannon *et al*. [29]	R/D	4,459 (5 years) 18%	45%	First ABG	24-hour	NR ≈ 6%	No	In-hospital death	Increased hospital mortality for every 100 mm Hg increase in PaO_2_
								Neurological function	
Bellomo *et al*. [31]	R/D	12,108 (10 years) 11%	68%	Worst (A-a) ΔO_2_ > 300 mm Hg	24-hour	NR ≈ 33%	No	In-hospital death^b^	Hyperoxemia did not affect outcome when adjusted for several confounders.
Janz *et al*. [32]	R	170 (5 years) ≈ 25%	80%	Highest PaO_2_	24-hour	Yes	No	In-hospital death	Increased hospital mortality for every 100 mm Hg increase in PaO_2_
								In-hospital CPC	
Ihle *et al*. [33]	R	584 (5 years) ≈ 6%	100%	Worst (A-a) ΔO_2_ > 300 mm Hg	24-hour	NR	No	In-hospital death	Hyperoxemia did not affect outcome.
Lee *et al*. [34]	R	213 (4 years) <3%	83%	Mean PaO_2_ value	24-hour	Yes	No	In-hospital death	V-shaped association between the mean PaO_2_ and poor neurologic outcome at hospital discharge
Vaahersalo *et al*. [35]	P	409 (1 year)	100%	Mean PaO_2_ value >300 mm Hg	24-hour	Yes (71%)	No	1-year CPC	PaO_2_ was not correlated to outcome

^a^Identification of an arterial oxygen pressure (PaO_2_) threshold to accurately separate patients with good and poor outcome. ^b^After adjustment on Acute Physiology and Chronic Health Evaluation III (APACHE III) score. (A-a)ΔO_2_, alveolo-arterial oxygen difference; ABG, arterial blood gas (analysis); CA, cardiac arrest; CPC, Cerebral Performance Category; CPR, cardiopulmonary resuscitation; FiO_2_, inspired oxygen fraction; HO, hyperoxemia; NA, not available; NR, not reported; NSE, neuron-specific enolase; OHCA, out-of-hospital cardiac arrest; P, prospective; R, retrospective; RCT, randomized clinical trial; R/D, retrospective analysis of database; ROSC, return of spontaneous circulation; S100B, protein S100B; TH, therapeutic hypothermia.

Few clinical studies have evaluated the role of hyperoxemia during the ICU stay on outcome of CA patients. Kilgannon and colleagues published two different analyses on the same large database from a cohort of patients from 131 US hospitals (Increase Minority Participation and Awareness of Clinical Trials (IMPACT) Database). In the first study [28], including 6,326 patients, hospital mortality was higher in patients with hypoxemia (defined as a PaO_2_ of less than 60 mm Hg or altered gas exchange with a PaO_2_/FiO_2_ ratio of less than 300) or hyperoxemia (PaO_2_ of more than 300 mm Hg) detected in the first arterial blood gas (ABG) available within 24 hours after admission, compared with patients with normal oxygen levels (hypoxemia 53% versus hyperoxemia 67% versus normoxemia 45%, *P* < 0.001). In a multivariable logistic regression analysis, hyperoxemia was independently associated with in-hospital mortality: odds ratio (OR) 1.8, 95% confidence interval (CI) 1.5 to 2.2. In the second study, after exclusion of patients with hypoxemia, the investigators wanted to evaluate the association between PaO_2_, considered as a continuous variable, and in-hospital mortality [29]. In the 4,459 patients studied, the median PaO_2_ was 231 (interquartile range 149 to 349) mm Hg and in-hospital mortality was 54%. Multivariable analysis yielded a significant 6% increase in mortality for any PaO_2_ increase by 25 mm Hg (OR 1.06, 95% CI 1.05 to 1.07). Although these two studies support the hypothesis that hyperoxemia is associated with worse outcome after CA, some limitations must be acknowledged. First, only a small proportion of patients were treated with hypothermia. Second, the use of the PaO_2_/FiO_2_ ratio does not really reflect hypoxemia, because it is a surrogate of lung failure rather than of low arterial oxygen content. Third, data were not collected following the Utstein style [30] or corrected for severity of disease (for example, Acute Physiology and Chronic Health Evaluation III, or APACHE III). Finally, and even more importantly, only the first ABG was taken into account in the final analysis instead of a mean value over the first 24 hours, which might be more representative of real oxygen exposure during this crucial period.

In a large Australian database (Australian and New Zealand Intensive Care Society-Adult Patient Database, or ANZICS-APD), Bellomo and colleagues [31] evaluated the correlation between different PaO_2_ levels and hospital mortality in 12,108 patients. Although these authors used the same definitions of hypoxemia and hyperoxemia used in previous studies [28,29], they reported a lower percentage of patients with hyperoxemia (11% [31] versus 18% [28]) but still a higher mortality rate for patients with hyperoxemia (59%) and hypoxemia (60%) when compared with normoxic patients (47%). In a multivariable model including some major confounding factors, hyperoxemia was significantly associated with mortality (OR 1.2, 95% CI 1.1 to 1.6); however, in a Cox proportional hazards regression model, after adjustment for other relevant covariates (year of admission, treatment limitations, patient’s lowest glucose level in the first 24 hours, patient’s indigenous status, and hospital source from home), PaO_2_ was no longer statistically associated with poor outcome (hazard ratio 1.1, 95% CI 1.0 to 1.2; *P* = 0.20). Despite the limitation of a retrospective analysis, this study highlights that some confounders not taken into account in previous studies may have influenced the statistical association between high PaO_2_ and poor outcome. Importantly, other issues may further limit the power of these findings. The PaO_2_ from the ABG analysis associated with the worst alveolo-arterial oxygen difference was used, and this PaO_2_ correlated only fairly with the mean PaO_2_ (as shown in the analysis of a subgroup of patients) and may not have adequately reflected the exposure of patients to high oxygen concentrations. This observation was also suggested by the lower PaO_2_ values recorded in this study when compared with previous publications (112 versus 231 mm Hg) [29]. Also, no data on neurological outcomes were reported. Finally, as in previous studies, only a minority of patients underwent hypothermia, but cooling is considered to mitigate ROS production and possibly influence hyperoxemia-mediated brain injury [8].

The first article to include patients treated with hypothermia [32] showed that those with a poor outcome had higher PaO_2_ values than others (254 versus 198 mm Hg; *P* = 0.022). A multivariable regression model, including factors known to be associated with poor outcome after CA, confirmed an independent correlation of PaO_2_ with mortality (OR for a PaO_2_ increment of every 100 mm Hg above 54 mm Hg 1.48, 95% CI 1.03 to 2.01) and worse neurological outcome (OR 1.48, 95% CI 1.03 to 2.14) at hospital discharge; however, neither FiO_2_ nor APACHE III score was included in the final analysis. Unfortunately, no specific cutoff of PaO_2_ was identified to predict poor outcome, although a PaO_2_ of more than 228 mm Hg was associated with a lower likelihood of neurological recovery. More recently, Ihle and colleagues [33] reviewed 584 patients selected from the ANZICS-APD database, for whom variables could be found following the Utstein model [30]. Unadjusted in-hospital mortality did not differ across different PaO_2_ ranges (51% hypoxia, 41% normoxia, and 47% hyperoxia; *P* = 0.28). In a multivariable model including CA characteristics, neither hypoxemia nor hyperoxemia was an independent predictor of in-hospital mortality. In a retrospective cohort of 213 adult patients with CA, Lee and colleagues [34] reported that PaO_2_ obtained during the first 24 hours was not related to hospital mortality or neurological outcome. In a multivariable model adjusted for established confounding factors after CA, the authors showed a V-shaped relationship between PaO_2_ and neurological outcome, with the highest probability of good neurologic outcome at PaO_2_ around 130 mm Hg. Finally, in a prospective observational study (n = 409), Vaahersalo and colleagues [35] calculated the proportion of time spent in different oxygen categories (less than 75 mm Hg, 75 to 150 mm Hg, 150 to 225 mm Hg, and more than 225 mm Hg) during the first 24 hours after CA. The proportion of time spent with a PaO_2_ of more than 225 mm Hg was similar between groups, as was the association of time within different PaO_2_ categories and outcome. Mean and highest PaO_2_ values were higher in patients with good neurological outcome than in those with poor neurological outcome (120 versus 113 mm Hg and 173 versus 150 mm Hg, respectively). Interestingly, the proportion of patients with good neurological outcome was higher in patients with the combination of high mean PaO_2_ and PaCO_2_ values.

## Perspectives and conclusions

The International Liaison Committee on Resuscitation states that oxygen administration should be titrated to obtain an oxygen saturation of 94% to 96% after ROSC [36]. Thus, routine administration of an FiO_2_ of 100% is no longer recommended after CPR [36,37]; however, it still seems prudent to use 100% oxygen during CPR, although the impact of high PaO_2_ on survival needs to be further evaluated. Although some studies have suggested that hyperoxemia after CA should be considered a cost-free, potentially modifiable risk factor for poor outcome, many potential confounders have been identified and strongly challenge this concept. Moreover, it is very difficult to identify a specific threshold of toxicity, as most of the studies used a PaO_2_ of more than 300 mm Hg to define hyperoxemia, while some brain injury may also potentially occur at lower values. Also, when a single ABG value was used, the proportion of patients with hyperoxemia ranged from 3% to 25% in the different studies [27-29,31-34]. Nevertheless, when all ABG values are considered, the incidence of hyperoxemia may exceed 40% [38], so that the real impact of exposure to high oxygen concentrations has probably been underestimated. Other parameters obtained from the ABG, such as acidemia or hypocapnia, may also be important determinants of poor outcome after CA [39,40]; however, these variables were not considered in these studies. Importantly, it is worthwhile to prospectively evaluate the mechanisms of oxygen toxicity after CA, such as seizure development, ROS production, impaired microcirculation, or the development of organ dysfunction. Finally, further studies are needed to help understand whether an absolute PaO_2_ value (that is, ‘peak’) could be more detrimental than a continuous exposure above a specific threshold and to assess the impact of the timing of hyperoxemia occurrence (early versus late phase after arrest) or of variability in oxygen levels (that is, from hypoxemia to hyperoxemia) in this setting.

## Key messages

- Hyperoxemia may potentially exacerbate or aggravate brain injury after experimental cardiac arrest.
- Hyperoxemia has been associated with controversial results in humans.
- Administering high FiO_2_ (100%) during CPR still seems to be advisable because it may facilitate ROSC.
- Because of the limited benefit of maintaining potentially harmful supra-normal oxygen levels in such patients, mechanical ventilation should be titrated to maintain an oxygen saturation between 94% and 96% in most patients after ROSC.

## Abbreviations

ABG: Arterial blood gas
ANZICS-APD: Australian and New Zealand Intensive Care Society-Adult Patient Database
APACHE III: Acute Physiology and Chronic Health Evaluation III
CA: Cardiac arrest
CI: Confidence interval
CPR: Cardiopulmonary resuscitation
FiO_2_: Inspired oxygen fraction
MOF: Multiple organ failure
NSE: Neuron-specific enolase
OR: Odds ratio
PaO_2_: Arterial oxygen pressure
PCAS: Post-cardiac arrest syndrome
ROS: Reactive oxygen species
ROSC: Return of spontaneous circulation
TTM: Target temperature management
